# Initial report on feasibility of PET/CT-based image-guided moderate hypofractionated thoracic irradiation in node-positive non-small cell lung Cancer patients with poor prognostic factors and strongly diminished lung function: a retrospective analysis

**DOI:** 10.1186/s13014-019-1304-2

**Published:** 2019-09-04

**Authors:** Chukwuka Eze, Julian Taugner, Olarn Roengvoraphoj, Nina-Sophie Schmidt-Hegemann, Lukas Käsmann, Cherylina Wijaya, Claus Belka, Farkhad Manapov

**Affiliations:** 1 Department of Radiation Oncology, University Hospital, LMU Munich, Marchioninistrasse 15, 81377 Munich, Germany; 2 Comprehensive Pneumology Center Munich (CPC-M), Member of the German Center for Lung Research (DZL), Munich, Germany; 3 German Cancer Consortium (DKTK), partner site Munich, Munich, Germany; 4 Department of Pulmonology, Asklepios-Fachkliniken München-Gauting, Munich, Germany

**Keywords:** Hypofractionated radiotherapy, Image-guided radiotherapy, NSCLC, Thoracic radiotherapy

## Abstract

**Background:**

To determine the feasibility of PET/CT-based image-guided moderate hypofractionated thoracic irradiation (Hypo-IGRT) in locally advanced node-positive non-small cell lung cancer patients with highly compromised pulmonary function.

**Method:**

Eight highly-selected and closely monitored patients with highly diminished pulmonary function (FEV1 ≤ 1.0 L and/or DLCO-SB ≤ 40% and/or on long-term oxygen therapy) were treated with Hypo-IGRT. Planning was based on 18F-FDG-PET/CT and 4D-CT in the treatment position. Hypo-IGRT was delivered to a total dose of 45 Gy (ICRU) in 15 daily fractions under strict image-guidance. Vital capacity (VC), forced expiratory volume in 1 s (FEV1), and single-breath diffusing capacity of the lung for CO (DLCO-SB) were analyzed prior to, 3 and 6 months after Hypo-IGRT.

**Result:**

Eight patients with stage IIIA-C NSCLC (8th TNM Ed.) completed Hypo-IGRT. The median follow-up was 29.4 months. The median age was 64 years. Four, three and one patient(s) presented with COPD GOLD IV, III and II, respectively and 5 patients (63%) were on long-term oxygen therapy. The median PTV was 226.9 cc (range: 100.17–379.80 cc). Median PFS and OS were 19 and 34.3 months. The 6 months and 1-year OS rates were 100, 87.5%, respectively. The 6- and 12- months PFS rates were 87.5 and 52.5%. Three patients developed local failure. Median initial VC, FEV1 and DLCO-SB was 1.69 L/64.8% predicted (range: 1.36–2.66 L/33–80%), 1 L/39.4% predicted (range:0.78–1.26 L/28–60% predicted) and 33.3% (range: 13.3–54%) predicted, respectively. Median values for VC, FEV1, DLCO-SB 3 and 6 months after Hypo-IGRT were 2.05 L/56.35% predicted (range: 1.34–2.33 L/47–81.5%), 1.08 L/47.5% predicted (range: 0.74–1.60 L/30.8–59.59%), 38.55% (range: 24–68%) and 1.64 L/66% predicted (range: 1.41–2.79/35.5–75.5%), 1.0 L/47% predicted (range: 0.65–1.28 L/24.5–54.10%), 31% (range: 27–43%), respectively. Mean lung dose was 9.4 Gy (range: 5.3–11.6 Gy) and V20 for both lungs was 15% (range: 6–19%). Mean esophageal dose was 12.76 Gy (range: 2.1–26.7 Gy). There was no case of grade 2 or higher radiation pneumonitis. Four patients developed grade 2 radiation esophagitis.

**Conclusion:**

Hypo-IGRT can be considered for individual and closely monitored patients with locally advanced node-positive NSCLC with highly compromised pulmonary function. No severe pulmonary toxicity and significant decline of pulmonary function parameters was observed in our cohort. Currently, this protocol is being assessed in an ongoing single-centre prospective study.

## Introduction

The contemporary standard of care for stage IIIA-C NSCLC consists of surgery for medically operable sub-classifications based on the Robinson criteria for stage IIIA disease or concurrent chemoradiotherapy (CRT) followed by consolidation therapy with Durvalumab with markedly improved survival rates based on the results of the PACIFIC trial [1].

CRT is often associated with increased acute and chronic toxicity, specifically pneumonitis and esophagitis [2]. In patients with poor performance status, CRT is not feasible as possible treatment-related side effects outweigh potential benefit. Additionally, patients with node-positive disease will often present with general comorbidities and impaired pulmonary function. High-risk patients with an FEV1 ≤ 1 L or DLCO-SB ≤ 40% predicted or on long-term oxygen therapy are unsuitable candidates for concurrent or sequential CRT and thus receive at best palliative treatment or best supportive care (BSC).

Stereotactic ablative radiotherapy (SABR) is an established treatment modality for early-stage NSCLC due to highly conformal delivery of high-dose radiotherapy to tumors whilst sparing adjacent organs at risk (OARs) and a proven very high local control rate of over 90% [3, 4]. Several studies have reported on the feasibility of hypofractionated radiotherapy in locally advanced (LA-) NSCLC demonstrating respectable results. However, these studies generally failed to include patients with poor performance status and highly compromised pulmonary function (see Table 1) [6, 7, 9–12]. We previously published a case report of 2 such patients successfully treated at our institution [13]. In this retrospective study, we present a follow-up analysis of eight such high-risk patients.

**Table 1:** Studies examining hypofractionation protocols

Study	PTV median	D total lung	D oesophagus	D heart	Pneumonitis	Esophagitis
Amini et al. 2012 [5]	–	–	–	–	≥ G2: 14 (11.8%)	≥ G2: 12 (10.1%)
Agolli et al. 2015 [6]	180 cc (73-682 cc)	(α/β = 3Gy) V20 < 25–30%, Mean dose ≤15Gy	(α/β = 3Gy) V42 < 32%	(α/β = 3Gy) V33 < 25%	Acute: G3–4 (6%) Late: G3–2 (3%) °4/5: 0	Acute: G3–3 (5%) Late: G3–1 (2%) G4/5: 0
Westover et al. 2015 [7]	50Gy: 673 ± 75 cc 55Gy: 646 ± 96 cc 60Gy: 454 ± 68 cc	For all dose steps: V18 < 37% Mean dose < 18Gy 1500 cc 15.5Gy 1000cc 16.3Gy	< 5 cc 51.3Gy Max. dose: 55.3Gy	< 15 cc 39.5Gy Max. dose: 48.9Gy	50Gy: 5 (33.3%) 55Gy: 4 (19.0%) 60Gy: 4 (21.1%)	50Gy: 0 55Gy: 1 (4.8%) 60Gy: 1 (5.3%)
Swanick et al. 2015 [8]	421.2 cc (46.6–2666.6 cc)	V20 < 30% Mean dose <20Gy	Mean dose <34Gy Max dose: 63.8Gy	Mean Dose <20Gy	G3–1 (1.4%) G4/5: 0	G3–3 (4.2%) G4–0 G5–1 (1.4%)
Pollom et al. 2016 [9]	139 cc (20-746 cc)	V18 < 37% MLD < 18 Gy 1500 cc 15.5Gy 1000cc 16.3Gy	< 5 cc 51.3Gy Max. dose: 55.3Gy	< 15 cc 39.5Gy Max. dose: 48.9Gy	4 (9.5%) [1 G4 Bronchiopleural fistula]	5 (11.9%)

**Table 2:** Treatment characteristics

PTV	D total lung	D oesophagus	D heart
Mean: 232.2 cm^3^	V20: 15% (range: 6-19%)	Mean: 12.76 Gy	Mean: 5 Gy
Median: 226.9 cm^3^	Mean: 9.4 Gy	(range: 2.1-26.7 Gy)	(range: 0.4-10 Gy)
(range: 100.2-379.8 cm^3^)	(range: 5.3-11.6 Gy)		

## Material and methods

### Patient cohort

Following institutional review board approval, we retrospectively reviewed the medical charts of 8 patients with histologically proven stage IIIA-C NSCLC (UICC 8th edition), poor performance status (ECOG-PS ≥ 2) treated between 04/14 and 06/17 and were not eligible for surgical resection, conventional radiotherapy or concurrent CRT based on their highly compromised pulmonary function defined as FEV1 ≤ 1.0 L and/or DLCO-SB ≤ 40% and/or on long-term oxygen therapy and registered comorbidities and thus underwent hypofractionated image-guided radiotherapy (Hypo-IGRT) to a total of 15 fractions in 3 Gy single fractions (BED_10_ = 58.5 Gy; α/β = 10 Gy) at our institution. All patients were discussed at the multidisciplinary tumor board following adequate staging via positron emission tomography with 2-deoxy-2-[fluorine-18] fluoro-d-glucose integrated with computed tomography (18F-FDG-PET/CT), endobronchial ultrasound with sampling and contrast-enhanced MRI of the brain as is common practice at our center. All patients received a radiotherapy dose adhering to a mean lung dose of less than 20 Gy, calculated from lung dose distributions corrected to equieffective doses at 2 Gy per fraction (EQD2) using the linear-quadratic model and an α/β of 2.5 Gy and the NTCP model in accordance with Kong et al. [14, 15] and normal tissue dose-volume constraints of other organs based on RTOG 0937 [16] in compliance with NCCN recommendations [17].

**Fig. 1:**
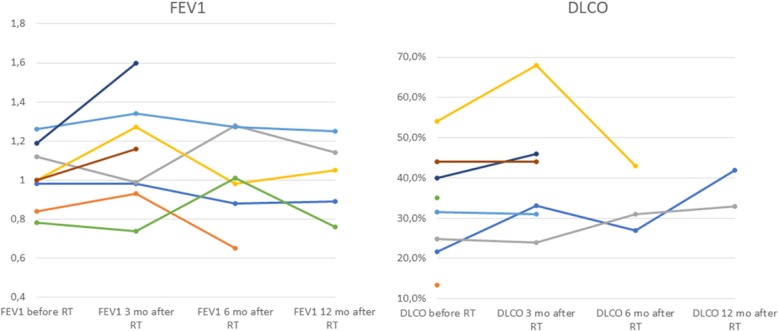
Line chart of available PFT values (FEV1 and DLCO-SB)

The patients were monitored closely during the course of radiotherapy and routinely underwent computed tomography (CT) of the chest and upper abdomen or 18F-FDG-PET/CT follow-up examination every 3 months after completion of radiotherapy and repeat imaging studies and clinical examinations ensuing every 3 months for the first 2 years, biannually in the following 2 years and annually from year 5 onwards. Lung function was assessed by pulmonary function tests (PFT) evaluating VC, FEV1 and DLCO-SB every 3 months for the first year following Hypo-IGRT and was assessed when available (see Fig. 1).

### Hypo-IGRT

Hypo-IGRT was performed following induction chemotherapy in 3 patients and as definitive treatment in 5. All patients underwent 18F-FDG-PET/CT and four-dimensional computed tomography (4D-CT) (10 sequential scans in all respiratory phases) in the treatment position immobilized in a vacuum-based and alpha cradle immobilization system.

The 4D-CT was matched with PET/CT for better definition of the target volume. The range of tumor motion was 1–4 mm in every dimension. Gross tumor volume (GTV) included the primary tumor and involved lymph node(s) (short axis ≥ 1 cm and/or PET-positive) post-induction chemotherapy in the respective patients. The internal target volume (ITV) was defined through the overlap of GTVs delineated on 10 phases of 4D-CT. Clinical target volume was not generated to limit pulmonary exposure. An isotropic margin of 5 mm was added to ITV to generate the planning target volume (PTV). The treatment was calculated on 3 mm grid with Collapse-Cone algorithm. Total dose delivered to the PTV was 45 Gy in 15 fractions, 5 times per week and was prescribed per ICRU with corrections for lung inhomogeneity. Pulmonary dose-volume parameters were calculated with total lung volume minus PTV. Mean lung dose (MLD), averages of ipsilateral and contralateral lung doses were obtained. Radiotherapy was delivered by linear accelerator (Elekta Synergy/Versa HD, Stockholm, Sweden) using 6 MeV 8–9 coplanar fixed photon beams. Image guidance was performed with kilovoltage-conebeam CT prior to each treatment. Toxicity monitoring was performed twice weekly to record any side effects. Dose constraints for the OARs (i.e., esophagus, spinal cord, lung, heart/pericardium etc.) were adopted from universal survival models in addition to published tissue properties.

### Treatment tolerability

Patients were closely monitored, and toxicity was graded according to the Common Terminology Criteria for Adverse Events (v4.03).

### Statistical analysis

Statistical analysis was performed using IBM SPSS software version 24 (IBM, New York, USA). Overall and progression-free survival were calculated from the date of initial pathological diagnosis. Kaplan-Meier survival analysis and log-rank test were used to evaluate progression-free and overall survival.

## Results

Eight patients completed Hypo-IGRT. The median age was 64 years. Four, three and one patients presented with stage IIIA, IIIB and IIIC (8th TNM Edition), respectively. However, the patient who presented ab initio with stage IIIC disease died 7 months after initial diagnosis. Seven patients were assigned an Eastern Cooperative Oncology Group Performance Score (ECOG PS) 2 and 1 patient ECOG PS 3. Four, three and one patient(s) presented with COPD GOLD IV, III and II, respectively and 5 patients (63%) were on long-term oxygen therapy. Three patients received chemotherapy prior to Hypo-IGRT. The median PTV was 226.9 cc (range: 100.2–379.8). Median initial VC, FEV1 and DLCO-SB was 1.69 L/64.8% predicted (range: 1.36–2.66 L/33–80%), 1 L/39.4% predicted (range:0.78–1.26 L/28–60% predicted) and 33.3% (range: 13.3–54%) predicted, respectively. Median values for vital capacity (VC), FEV1, DLCO-SB 3 and 6 months after Hypo-IGRT were 2.05 L/56.35% predicted (range: 1.34–2.33 L/47–81.5%), 1.08 L/47.5% predicted (range: 0.74–1.60 L/30.8–59.59%), 38.55% (range: 24–68%) and 1.64 L/66% predicted (range: 1.41–2.79/35.5–75.5%), 1.0 L/47% predicted (range: 0.65–1.28 L/24.5–54.10%), 31% (range: 27–43%), respectively (see Fig. 1). PFT values for some patients were not available at various time points during follow-up. Mean lung dose was 9.4 Gy (range: 5.3–11.6 Gy). V15 and V20 for both lungs were 22% (range: 10–25%) and 15% (range: 6–19%), respectively (see Table 2).

**Fig. 2:**
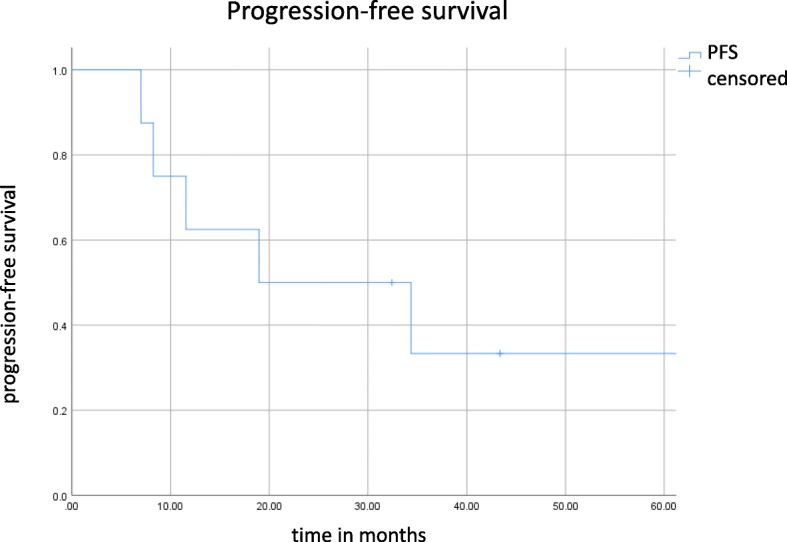
Kaplan-Meier curve for progression-free survival. Tick marks indicate censoring

**Fig. 3:**
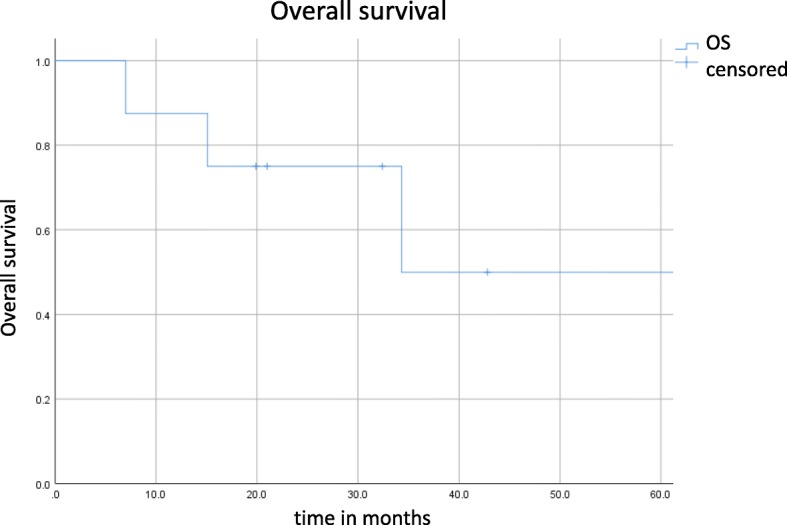
Kaplan-Meier curve for overall survival. Tick marks indicate censoring

The median follow-up was 29.4 months. Three patients developed local failure. The median PFS and OS were 19 and 34.3 months (see Figs. 2 and 3), respectively. The 6 months and 1- and OS rates were 100 and 87.5%, respectively. The 6- and 12- months PFS rates were 87.5 and 52.5%, respectively. Mean esophageal dose was 12.76 Gy (range: 2.1–26.7 Gy). There was no case of ≥ grade 2 radiation pneumonitis. However, 4 patients developed grade 2 radiation esophagitis.

## Discussion

Since mid- to end of the 1990s, several studies have demonstrated feasibility of moderate hypofractionated thoracic radiation for node-positive LA-NSCLC with a variety of prescription doses ranging from 45 in 15 fractions to 75 in 28 fractions and differing outcomes regarding survival and toxicity [18]. Two previous studies examined in the abovementioned systematic review also included node-positive stage III patients with poor initial performance status. Nguyen et al. reported equivalence of moderate hypofractionated thoracic irradiation (45 Gy in 15 fractions) to conventional treatment regimens (60-66 Gy in 30–33 fractions) in LA-NSCLC patients with borderline prognostic factors despite the fact that the patients in the hypofractionation group had inferior prognostic factors [5]. In a follow-up analysis by the same group, these findings were consolidated and demonstrated equipoise regarding local or distant control as well as overall survival compared to the standard normofractionated radiatiotherapy arms [10]. Accelerated hypofractionated protocols were associated with improved patient compliance and were more cost-effective.

Based on our prodigious literature research, we determined the 45 Gy in 3 Gy single fractions protocol to be the most appropriate and applicable in node-positive stage IIIA-B NSCLC patients with poor PS and strongly diminished lung function (FEV1 ≤ 1 L or DLCO ≤40% predicted) as these patients are not candidates for conventional treatment modalities and are thus condemned to palliative chemotherapy, radiotherapy alone or BSC associated with dismal prognosis [19].

We screened our retrospective institutional database to identify a corresponding cohort. Eight highly selected and closely monitored patients with node-positive stage III A-C disease with initial PS ECOG 2–3, extensive comorbidities and highly compromised pulmonary function were included in the retrospective analysis. All patients completed initial diagnostic work-up including contrast-enhanced cranial MRI and received Hypo-IGRT at our centre with a documented follow-up. Hypo-IGRT was applied according our in-house protocol. Importantly, obligatory PET/CT and 4D-CT-based treatment planning including omission of the CTV and daily image-guidance during radiation delivery were obligatory [13].

Our results demonstrated with a durable median follow-up of 29.4 months that meticulously planned and monitored Hypo-IGRT can be considered in this high-risk subgroup. 6-, 12- and 18-month overall survival rates were encouraging. In addition, 12-month PFS was comparable to previous data from the University of Texas MD Anderson Cancer Centre group and fared favourably compared to conventionally treated patients [5, 10]. Furthermore, particular emphasis must be made to the low rate of acute toxicity despite in the current study. Only one patient (12.5%) had a 4-day treatment interruption because of exacerbation of pre-existing severe COPD Gold IV and was hospitalized. No case of >G2 radiation pneumonitis was documented.

As a result of various risk factors i.e. poor performance status and highly reduced pulmonary function, moderate hypofractionation with a total tumor dose of 45 in 3 Gy single fractions (corresponding BED_10_ 58.5 Gy; α/β = 10) was preferred to conventional treatment regimens. Nevertheless, 6- and 12 months local control rates 87.5 and 52.5% were achieved. Based on previous studies, no significant positive effect of tumor dose escalation over BED_10_ 60 Gy (α/β = 10) on overall survival in this high-risk patient subgroup exists [10].

Based on the analysis by Kaster et al., the relationship between BED and survival is moderately strong and demonstrated a moderate linear relationship between lesional BED and overall survival in the order of an absolute survival benefit up to a maximum 0.7% for every 1 Gy increase including moderate hypofractionation protocols.

The evaluation of PFT parameters did not demonstrate significant decline in VC, FEV1 nor DLCO-SB. These results are generally in accordance with previously reported on SABR for predominantly medically inoperable early-stage NSCLC [11, 12]. Stone et al. demonstrated although statistically significant, nearly all declines in PFTs as G1 on the RTOG scale and PFT declines to not be associated with inferior survival [11]. Stanic and colleagues observed that poor baseline PFT did not predict pulmonary toxicity nor decreased overall survival after SABR and concluded that poor baseline PFT alone should not be used to exclude patients with early stage lung cancer from treatment with SABR [12]. This formed the basis for our rationale in treating the patients in the current study. Poor initial PS and strongly diminished pulmonary function did not seem to predict excessive pulmonary toxicity nor inferior survival.

Certainly, confirmation of these results in a prospective setting enrolling a larger number of patients is absolutely imperative. As such, we initiated a single-centre study investigating this promising protocol in a previously neglected patient cohort.

Acknowledging the weaknesses of the current analysis, the small sample size cannot be overlooked as well as the inherent shortcomings of its retrospective nature. Nevertheless, to the best of our knowledge, this is the first analysis addressing feasibility of Hypo-IGRT in this high-risk patient cohort with poor performance status and diminished lung function. Indeed, some might question our rationale and might deem this protocol as highly risky, however our results could dispel such claims as no grade 3, 4 and 5 toxicities were reported.

## Conclusion

Hypo-IGRT can be considered for individual and closely monitored patients with locally advanced node-positive NSCLC with highly compromised pulmonary function. No severe pulmonary toxicity and significant decline of pulmonary function parameters was observed in our cohort. Currently, this protocol is being assessed in an ongoing single-centre prospective study.

## Data Availability

The datasets used and analysed during the current study are available from the corresponding author on reasonable request.

## Abbreviations

18F-FDG-PET/CT: Positron emission tomography with 2-deoxy-2-[fluorine-18] fluoro-d-glucose integrated with computed tomography
4D-CT: Four-dimensional computed tomography
BED: Biologically effective dose
BSC: Best supportive care
COPD: Chronic obstructive pulmonary disease
CRT: Chemoradiotherapy
CT: Computed tomography
CTV: Clinical target volume
DLCO-SB: Single-breath diffusing capacity of the lung for CO
ECOG: Eastern Cooperative Oncology Group
FEV1: Forced expiratory volume in 1 s
GTV: Gross tumour volume
Hypo-IGRT: Hypofractionated thoracic irradiation
IBM: International Business Machines Corporation
ICRU: International Commission on Radiation Units & Measurements
ITV: Internal target volume
LA: locally advanced
MLD: Mean lung dose
MRI: Magnetic Resonance Imaging
NSCLC: Non-small cell lung cancer
OAR: Organs at risk
OS: Overall survival
PFS: Progression-free survival
PFT: Pulmonary function tests
PS: Performance score
PTV: Planning target volume
RTOG: Radiation Therapy Oncology Group
SABR: Stereotactic ablative radiotherapy
SPSS: Statistical package for the Social Sciences
TNM: Tumor-Node-Metastasis
V15: The volume of normal lung receiving 15 Gy
V20: The volume of normal lung receiving 20 Gy
VC: Vital Capacity
α/β: alpha/beta ratio
